# Supplementary material for: A comparison of two common sample preparation techniques for lipid and fatty acid analysis in three different coral morphotypes reveals quantitative and qualitative differences
Source: PeerJ. 2017 Aug 2;5:e3645. doi: 10.7717/peerj.3645 (PMC5544933; doi:10.7717/peerj.3645)
Supplement: Supplemental Information 1 — Table S1 - Lipid class composition of intact samples of four scleractinian species prepared with crushing method. Table S2 - Major fatty acid class composition of intact samples of four scleractinian species prepared with crushing method. [file peerj-05-3645-s001.docx]

### *Supplementary Table 1*

Lipid class composition of intact samples of four scleractinian species prepared with crushing method.

| *(mg g lipid^-1^)* | ***A. millepora*** | ***M. crassotuberculata*** | ***P. cylindrica*** | ***P. damicornis*** |
| --- | --- | --- | --- | --- |
| **Wax ester** | 123±15.9 | 85.9±11.1 | 88.1±6.4 | 95.6±7.2 |
| **Triacylglycerol** | 194±78.4 | 170±76.1 | 70.7±12.7 | 189±63.5 |
| **Free fatty acid** | 41.8±7.6 | 21.5±4.4 | 36.1±26 | 45.8±7.4 |
| **1,2-diacylglycerol** | 74.2±18 | 97.1±12.2 | 112±5 | 82.2±19.5 |
| **Sterol** | 80.1±10.4 | 74.7±9 | 79.2±10.6 | 71.7±7 |
| **AMPL** | 175±26.2 | 173±36.1 | 171±16.2 | 134±13.8 |
| **Phosphatidylethanolamine** | 84.4±16.7 | 118±24.1 | 120±4.1 | 99±9.4 |
| **Phosphatisylserine-Phosphatidylinositol** | 104±35.3 | 115±43 | 153±32.9 | 122±35.8 |
| **Phosphatidylcholine** | 123±17.8 | 133±20.3 | 151±7 | 123±14.6 |
| **Lysophosphatidylcholine** | 0±0 | 12.1±15.1 | 19.1±21.4 | 38.1±23.3 |
| **∑STORAGE** | 433±78.1 | 375±78.9 | 307±23.9 | 412±71 |
| **∑STRUCTURAL** | 487±69.8 | 551±75.5 | 614±29.1 | 516±67.3 |

Values are presented as means ±SEM (n=20).

### *Supplementary Table 2*

Major fatty acid class composition of intact samples of four scleractinian species prepared with crushing method.

| *(mg g lipid^-1^)* | ***A. millepora*** | ***M. crassotuberculata*** | ***P. cylindrica*** | ***P. damicornis*** |
| --- | --- | --- | --- | --- |
| **Total** | 349±85.2 | 300±97.8 | 334±94.2 | 258±103 |
| **SFA** | 174±53 | 165±73.4 | 144±47.9 | 138±56.8 |
| **MUFA** | 44.4±12 | 32.3±9.44 | 47.1±13.6 | 41±19.3 |
| **PUFA** | 100±10.9 | 92.1±14.7 | 130±33.9 | 65.1±24 |
| **n-3 PUFA** | 56.4±6.3 | 22.3±3.7 | 65.6±19.4 | 32.3±13.1 |
| **n-6 PUFA** | 43.5±5.08 | 68.6±11.4 | 63.6±17 | 32.6±11.5 |

Values are presented as means ±SEM (n=20).
